# Dual stimulation in unexpected poor responder POSEIDON classification group 1, sub–group 2a: A cross-sectional study

**DOI:** 10.18502/ijrm.v13i6.7287

**Published:** 2020-06-30

**Authors:** Maryam Eftekhar, Banafsheh Mohammadi, Parisa Khani, Maryam Mortazavi Lahijani

**Affiliations:** ^1^Department of Obstetrics and Gynecology, Research and Clinical Center for Infertility, Yazd Reproductive Sciences Institute, Shahid Sadoughi University of Medical Sciences, Yazd, Iran.; ^2^Abortion Research Center, Yazd Reproductive Sciences Institute, Shahid Sadoughi University of Medical Sciences, Yazd, Iran.; ^3^Department of Obstetrics and Gynecology, Rafsanjan University of Medical Sciences, Rafsanjan, Iran.

**Keywords:** Dual stimulation, Poor responder, POSEIDON classification, Luteal-phase, Follicular phase, Ovarian stimulation.

## Abstract

**Background:**

Poor ovarian response management is a complex and controversial issue in the field of reproductive medicine.

**Objective:**

The aim of this study was to apply double stimulation in the same cycle in unexpected poor responders in POSEIDON classification group 1, sub group 2a and compare assisted reproductive technology outcomes between luteal phase and follicular phase ovarian stimulation.

**Materials and Methods:**

In this cross-sectional study, 10 women with age < 35 yr, antral follicle count > 5, and anti-müllerian hormone > 1.2 ng/mL were enrolled. All participants received conventional antagonist protocol in the follicular phase and only the cycles with retrieved oocytes < 4 in this phase included. The luteal phase ovarian stimulation was initiated from the day of first oocytes retrieval by 300 IU of human menopausal gonadotropin / day. When dominant follicles amounted to 14 mm in mean diameter, 0.25 mg/day of gonadotropin-releasing hormone antagonist was initiated and 10,000 IU human chorionic gonadotropin was injected when at least two follicles with a mean diameter of 17 mm were observed. Oocyte retrieval was carried out 34-36 hr following human chorionic gonadotropin injection. Finally, a comparison was made between the two phase in terms of the number of retrieved oocytes as well as the number of obtained embryos and fertilization rates.

**Results:**

Numbers of retrieved oocytes (p = 0.004), mature oocytes (p = 0.016), and embryos (p = 0.013) was significantly higher in luteal phase in compared with follicular phase. Quality of embryos was similar in two phases.

**Conclusion:**

Double stimulation protocol can increase number of retrieved oocytes in unexpected PORs

## 1. Introduction 

Poor ovarian response in women treated with in vitro fertilization (IVF) is one of the major challenges in this field. The incidence of poor ovarian response in recent studies is estimated to be 9-24% of women undergoing IVF undergoing IVF (1). However, the clinical management of poor ovarian responders (PORs) still remains a matter of controversy. Researchers have recommended several strategies to manage PORs, but the fact remains that there is no general agreement on the best treatment protocol for each subgroup. There is no suitable treatment protocol for unexpected PORs during the ART cycles (2). The concept of unexpected PORs is usually attributed to the patients with normal ovarian reserve aged < 35 years and poor ovarian responses to controlled ovarian hyperstimulation (COH) and few retrieved oocytes (3).

To reduce the heterogeneity of PORs, the new POSEIDON classification (Patient-oriented Strategies Encompassing Individualize D Oocyte Number) was developed to provide a higher detailed classification of these patients (4). This classification has distinguished between those who unexpectedly respond poorly to exogenous stimulation and those whose response to stimulation is invariably poor, that is, between good and poor ovarian reserve patients, respectively. It is based on age, ovarian biomarkers (i.e., antral follicle count (AFC) and anti-müllerian hormone (AMH)), and previous ovarian response (5). On the basis of POSEIDON stratification, group 1, sub-group 2a comprised of young patients aged < 35 yr with adequate pre-stimulation ovarian reserve parameters (AFC > 5, AMH > 1.2 ng/mL, oocyte < 4), and with an unexpected poor or sub-optimal ovarian response (6). For managing a poor responder, varying strategies have been suggested; such as increase the dose of FSH, recombinant luteinizing hormone supplementation and dehydroepiandrosterone supplementation before COH (2). Patients with low ovarian reserve, both in POSEIDON groups 3 and 4 need special attention in terms of pretreatment strategy, ovarian stimulation, adjuvant treatment, as well as ovulation trigger strategy. These strategies can optimize the likelihood of having at least one euploid blastocyst for transfer (7). The pathophysiological mechanisms explained the hyporesponse to gonadotropin stimulation have yet to be known. However, factors such as specific genotypic traits and environmental contaminants are supposed to contribute to the matter. Genetic polymorphisms affecting the gonadotropins and their receptors, in particular, may affect ovarian stimulation results. (8). The combination of conventional follicular and luteal phase stimulation is achieved by double stimulation in the same ovarian cycle (9). For infertile women, luteal phase ovarian stimulation (LPOS) has been identified as a feasible protocol. The high levels of progesterone in the luteal phase prevent the premature luteinizing hormone surge; this event is definitely beneficial for PORs (10). The aim of this study was to apply double stimulation in the same cycle in unexpected PORs in POSEIDON classification group 1, sub-group 2a and compare ART outcomes between LPOS and FPOS.

## 2. Materials and Methods 

In this cross-sectional study, we reviewed the records of women referred to Yazd Reproductive Sciences Institute, Yazd, Iran for infertility treatment from April to September 2019. All women aged < 35 yr with sufficient pre stimulation ovarian reserve parameters (AFC > 5, AMH > 1.2 ng/mL) were enrolled in the study. Our exclusion criteria were severe male factor, severe endometriosis, hydro salpinx, and the history of any endocrine disorders except PCOS.The GnRH antagonist protocol was used to treat all participants in the follicular phase by injecting 150 IU recombinant human follicle stimulating hormone (Cinal-F, Iran), subcutaneously. After five days, vaginal ultrasound was serially performed, and when a mature follicle (≥ 14 mm) was detected, a 0.25 mg/daily GnRH antagonists (Cetrotide (Cetrorelix); Merck Serono Laboratories, Aubonne, Switzerland) was subcutaneously injected. After observingleast two 17 mm-diameter follicles, 0.2 mg GnRH-a (Decapeptyl, Ferring Co., Germany) was injected subcutaneously as well. 36 hr later, transvaginal oocyte retrieval was performed under sedation. All embryos were morphologically evaluated on the second day after the ovarian puncture and accomplished to IVF or intra-cytoplasmic sperm injection (ICSI). In the cases with < 4 retrieved oocytes, the LPOS was initiated with 300 IU of HMG (Merional, IBSA, Lugano, Switzerland) on first retrieval day and embryos were vitrified. Serial vaginal ultrasound was performed to evaluate the ovarian response. GnRH antagonist (0.25 mg/day) was started when the dominant follicles reached 14 mm in mean diameter, and continued until the day of hCG injection. When at least two follicles with a mean diameter of 17 mm were observed, 10,000 IU hCGs (Pregnyl, Organon, the Netherlands) were injected and 34 to 36 hours after that, oocyte retrieval was carried out. Conventional IVF or ICSI were performed similar to conventional antagonist protocol, and all embryos were cryopreserved by vitrification method. Finally, a comparison was made between the two phases in terms of the number of retrieved oocytes as well as the number of obtained embryos and fertilization rates.

### Ethical consideration

This study was approved by the ethics committee of the Yazd Reproductive Sciences Institute, Shahid Sadoughi University of Medical Sciences, Yazd, Iran (IR.SSU.RSI.REC.1397.031).

### Statistical analysis

SPSS software version 15.0 for windows (Statistical Package for the Social Sciences, Chicago, IL, USA) was used for data analysis using the Student's *t* test and Chi-square test. P-value < 0.05 was considered as statistically significant.

## 3. Results

A total of 10 women who met our inclusion criteria were enrolled in the study (Table I). The number of retrieved oocytes, mature oocytes, and embryos was significantly higher in luteal phase in comparison with the follicular phase (p = 0.004, p = 0.016, and p = 0.013, respectively). The quality of embryos were similar between embryos obtained after follicular or luteal phase stimulation (p = 1.000) (Table II).

**Table 1:** Basic characteristics of the participants


**Variables**		**Outcome**
Age (yr) *		30.70 ± 3.40
Duration of infertility (yr) *		5.95 ± 3.13
AMH (IU/L) *		5.45 ± 2.80
BMI*		24.23 ± 1.31
Etiology of infertility**		
	**PCOS**	3 (30)
	**Mal factor**	2 (20)
	**Unexplained**	1 (10)
	**Mixed**	4 (40)
* Data presented as Mean ± SD. ** Data presented as n (%), AMH: Anti-mullerian hormone; BMI: Body mass index		

**Table 2:** Comparison of ART outcomes between the two study groups


**Variables**		**Follicular cycle**	**Luteal cycle**	**P-value**
**Retrieved oocytes **(1)*		1.9 ± 1.10	9.22 ± 6.81	0.004
**Mature oocytes **(1)*		1.70 ± 0.82	7.87 ± 5.02	0.016
**Embryo **(1)*		1.33 ± 0.51	4.85 ± 2.85	0.013
Quality of embryos**				
	**A**	33.3	28.6	1.000
	**B**	66.7	71.4	
Maturation rate**		94.17	78.12	0.038
Cleavage rate **		88	100	0.30
Fertilization rate **		90.48	53.75	0.009
Cycle duration (day)*		10.70 ± 3.16	8.50 ± 3.53	0.16
**Gonadotropin dose **(8)*		639.83 ± 202.33	1131 ± 357.77	0.070
Estradiol level in trigger day (pg/ml)*		572.50 ± 533	1884.90 ± 1668	0.029
*Data presented as Mean ± SD and analyzed by Student's *t* test, ** Data presented as %.and analyzed by Chi-square test				

## 4. Discussion

In this pilot study, we made attempts to show if dual stimulation can be considered a good option for the management of PORs classified as group 1, sub-group2a? Our results indicated a higher significant number of retrieved oocytes and embryos in luteal phase compared with follicular phase in these subgroups.

In a study by Kuang and co-workers, a mild ovarian stimulation was conducted on 38 women. They administered the human menopausal gonadotropin and letrozole to women after the first oocyte retrieval. When dominant follicles had matured, the second oocyte retrieval was performed. Their results showed that double ovarian stimulations in the same menstrual cycle provided more opportunities in poor responders (according to Bologna criteria) for oocyte retrieval. They concluded that the luteal phase stimulation can lead to more retrieved oocytes in the short time. (9).

According to our review of literature, this study is the first to compare follicular and luteal ovarian stimulation in an ovarian stimulation cycle in unexpected PORs. This study was different from others in starting ovarian stimulation in the day of first oocyte retrieval. The findings demonstrated that earlier onset of ovarian stimulation in the luteal phase fails to have a negative effect on ovulation stimulation results; it shortens the duration of stimulation as well as the patient's stay. For the inappropriate condition of the endometrium, all embryos were cryopreserved. Similarly, in a retrospective study conducted by Zhang, a comparison of the clinical outcomes of follicular and luteal phase and double ovarian stimulation in the patients with PORs (Bologna criteria) undergoing IVF was made, the results of which indicated longer stimulation, higher dosages of HMG, and higher MII oocyte rates in Luteal phase (p < 0.001). They did not report the significant difference in clinical pregnancy (CPR) and live birth rate (LBR) between the two groups. The number of oocytes retrieved in the luteal phase stimulation protocol was higher in the double ovarian stimulation group, however, the luteal phase stimulation showed a lower rate of MII oocytes (p = 0.031). In addition, there were no statistical differences between CPR and LBR (11).

Concerns have grown over the increased aneuploidy in embryos obtained during the luteal phase. In a study, Ubaldi and co-workers compared the euploid blastocyst formation rates in the follicular versus luteal phase in the same menstrual cycle between the patients with reduced ovarian reserve. Their results showed no signiﬁcant difference in the number of retrieved oocytes, MII oocytes, biopsied blastocysts per stimulated cycle, and also the euploid blastocyst rate calculated either per biopsied blastocyst or injected MII oocyte in the follicular vs luteal phase stimulation (12). Another study by de Almeida Cardoso and colleagues concluded that the double stimulation protocol significantly increased the number of retrieved oocytes and mature oocytes to be injected, but there were no significant differences in the fertilization and blastocyst rates (13).

## 5. Conclusion 

For generating an adequate number of oocytes, it stands to reason to accept that double stimulation is advantageous for patients with unexpected PORs. Accumulation of oocytes in a single cycle of stimulation that helps minimize the time in which it will be performed is the greatest benefit of this protocol. Moreover, it bears the potential to allow generating a larger number of embryos, which can then be genetically evaluated, thereby favoring the final clinical result.

##  Conflict of Interest

The authors declare that there is no conflict of interest.
